# Primary Immunodeficiency Diseases: Current and Emerging Therapeutics

**DOI:** 10.3389/fimmu.2017.00937

**Published:** 2017-08-09

**Authors:** Beatriz E. Marciano, Steven M. Holland

**Affiliations:** ^1^Laboratory of Clinical Infectious Diseases, National Institute of Allergy and Infectious Diseases (NIAID), National Institutes of Health (NIH), Bethesda, MD, United States

**Keywords:** immunodeficiency, immune modulation, chronic granulomatous disease, leukocyte adhesion deficiency, interferon gamma

## Abstract

Primary immunodeficiency diseases (PID) result from defects in genes affecting the immune and other systems in many and varied ways (1, 2). Until the last few years, treatments have been largely supportive, with the exception of bone marrow transplantation. However, recent advances in immunobiology, genetics, and the explosion of discovery and commercialization of biologic modifiers have drastically altered the landscape and opportunities in clinical immunology. Therapeutic options and life expectancy of PID patients have also improved dramatically, in large part as a result of better prevention and treatment of infections as well as better understanding and treatment of autoimmune complications (3). As early-life infection-related mortality declines we should anticipate the emergence of other conditions that were previously not appreciated, including malignancies and degenerative disorders unmasked by increasing longevity (4). The genomic revolution has identified literally hundreds of new genetic etiologies of immune dysfunction, many of which are or will soon be eligible for targeted therapies. These emerging immunomodulatory agents represent new therapeutic options in PIDs (5).

## Therapy Based on Clinical Practice

### Prophylaxis

Invasive bacterial, fungal, viral, and mycobacterial infections carry a high morbidity and mortality in the immunocompromised, and therefore, enormous effort has been directed at prevention. The advent of antibiotics in the last century was critical for the survival of patients with primary immunodeficiency disease (PID) (6). Even before the advent of antibiotic prophylaxis in advanced HIV infection, which transformed AIDS from a rapidly fatal to a more manageable disease, long-term prophylactic antibiotics were widely implemented in chronic granulomatous disease (CGD), with excellent effects (7). CGD also served as the first PID for which cytokine therapy with interferon gamma (IFNγ) (8) and antifungal prophylaxis was indicated and approved (9). The reasons that CGD played such a prominent role in the development and study of prophylaxis were that it was relatively survivable with medical management and bone marrow transplantation was relatively infrequent until recently. Therefore, a relatively large population of patients was able to participate in clinical trials, which markedly assisted the exploration and development of therapeutics. This paradigm has been missing from several other diseases, either because they are not as survivable or because bone marrow transplantation has been more aggressively practiced [e.g., severe combined immune deficiency, Wiskott–Aldrich syndrome (WAS), hyper-IgM syndrome]. For these reasons, many of the approaches discussed below will not have significant prospective clinical validation, making it necessary to rely upon mechanistic explanations and anecdotal reports. While it is likely that in the relatively near future treatments to replace (transplantation) or repair (gene therapy) the underlying B cell, T cell, and NK cell defects will be available, it is most likely that immunopathology will continue to be a prominent and recurring cause for PID patients to seek care.

The dual and somewhat conflicting demands of immune deficiency, with its recurrent and severe infections, and immune dysregulation, with its associated autoimmunity or autoinflammation, are now well appreciated as a hallmark of PID (10). This means that balancing close attention to infection susceptibility against needs for immunosuppression poses a major therapeutic challenge (11).

### Steroids

Corticosteroids have been the backbone of immune modulation since their discovery in the 1930s and their use in rheumatoid arthritis (RA) in 1948 (12, 13). Despite their long use, their long-term toxicity remains significant and their specific mechanisms poorly understood. However, they continue to be a mainstay and first-line approach to immune modulation, especially when needed urgently. Their broad, rapid onset of effects on all the major actors of immune response (T, B, NK, neutrophils) as well as nominally non-immune pathways (e.g., wound healing, glucose metabolism, adrenal suppression) make them both highly effective and very difficult to clearly understand. Their significant complications such as polyphagia, diabetes, cataracts, osteopenia, and infections limit their utility and keep provider and patient anxiety levels high.

Despite these concerns, corticosteroids effectively manage the hollow viscera obstructive and inflammatory disorders in CGD and are surprisingly well tolerated with minimal additional infectious complications at low doses (14). In CGD, steroids have also been used in the treatment of “mulch pneumonitis,” the acute inflammatory and necrotizing granulomatous lung disease that follows inhalation of organic matter, such as mulch, compost, dirt, or hay (15). In the CGD mouse model, fatal pulmonary granulomatous inflammation can be caused by either live *Aspergillus* hyphae, which cause invasive fungal infection, or, more surprisingly, by dead *Aspergillus* hyphae, which cause a severe fatal granulomatous response (16). Further, in CGD, steroids have been used in the setting of staphylococcal liver abscess, where they can largely obviate the need for surgery and better preserve long-term liver function (17). They have also helped with the management of necrotizing *Nocardia* pneumonias (18). Therefore, in CGD both invasive infection and hyper-inflammation vie as causes of morbidity and mortality and can be successfully managed with judicious coadministration of both steroids and antibiotics.

### Cytokine Therapy

#### Interferon Alpha (IFNα)

Defects in the TLR3 pathway have been clearly defined as causes of recurrent herpes simplex encephalitis (HSE) in children due to mutations in TLR3, UNC-93B, TRIF, TBK-1, TRAF-3, and IRF3 (19). All of these genes converge on the pathways for neuronal IFNα production, mutations in which put neurons at risk for uncontrolled herpesviral replication. Importantly, in some of these defects (*TBK1, TLR3, TRAF3*) peripheral blood mononuclear cell IFNα production is normal, even though fibroblast and neuronal production are impaired. Therefore, the search for defects involved in HSE should be done genetically and not based entirely on the *in vitro* responses found in peripheral blood samples. Exogenous IFNα or IFNβ therapy clearly rescue the viral phenotype *in vitro*, suggesting that IFNα or IFNβ therapy might be useful in cases of HSE associated with defects in the TLR3 pathway (20, 21). Whether IFNα therapy might have activity in cases of HSE without defects in the TLR3–IRF3 pathway is unclear at this point.

#### Interferon Gamma (IFNγ)

After its early cloning and production, expectations for the clinical applications of IFNγ were high. Unfortunately, very few of those expectations were realized. However, the observation that IFNγ increased superoxide production from monocyte-derived macrophages *in vitro* led to interest in using it in therapy for chronic granulomatous disease (CGD). An international, prospective, randomized double-blind trial in CGD patients showed clear reduction in severe infections in the IFNγ (50 μg/m^2^ subcutaneously three times weekly) group without exacerbation of granulomatous or inflammatory complications (22). IFNγ is essential for the killing of many intracellular microbes and has been used to enhance anti-mycobacterial immunity in patients with partial dominant IFNγ receptor 1 deficiency and chemotherapy-resistant mycobacterial infection (23). Higher doses of IFNγ (200 µg/m^2^) have been used in those with mycobacterial infections in the dominant partial forms of IFNγ receptor deficiency and in recessive IL12Rβ1 deficiency (24).

## Therapy Based on Mechanism

### Interleukin-2

Interleukin-2 is secreted from T cells and supports T cell proliferation, NK cell activation, and can promote activation-induced cell death (25, 26). However, at low does, recombinant IL-2 has also been shown to selectively increase T regulatory (Treg) cells. In the WAS, IL-2 therapy was recognized to enhance killing activity *in vitro* (27). In a prospective study WAS patients responded to low-dose IL-2 (0.5 MU/m^2^ for 5 days every 3 weeks) with modest increases in lymphocyte counts. However, consistent with the narrow dose range for many cytokines, at the 1 MU dose several patients had worsened thrombocytopenia (28). In the setting of bone marrow transplantation low-dose and very low-dose IL-2 have been shown to increase Treg cells, but their long-term value in preventing graft-versus-host disease (GVHD) is still being determined (29).

### Cell Depletion

#### CD 20

Rituximab (anti-CD20) is active in the treatment of lymphoma and in many autoimmune diseases, presumably through B cell depletion and also through disruption of autoantibody production. However, depletion of CD20+ B cells also removes potent antigen presenting cells, so it may have more than one mechanism of action. Improved quality of life has been observed in those with B-cell class-switch defects (hyper-IgM syndrome), who received rituximab for the treatment of autoimmune manifestations and generalized lymphadenopathy (30). Rituximab has been used as immunomodulatory therapy, especially as part of the treatment of granulomatous lymphocytic interstitial lung disease (31) in CVID as well as in CVID-associated autoimmune cytopenias (32–34). In patients with anti-IFNγ autoantibodies who have severe disseminated infections with intracellular pathogens, especially non-tuberculous mycobacteria, rituximab has helped to reduce antibody levels allowing clearance of infection (35).

### Cytokine Antagonists

#### Anti-IL-17 and Anti-p40 (Anti-IL-12/23)

IL-17 is an important mediator of inflammation, especially at epithelial surfaces. It is itself induced in CD4 + T cells by IL-23 and in turn induces the generation of G-CSF and IL-22. IL-17 neutralization has profoundly beneficial effects on the clinical courses of psoriasis and colitis (36). IL-23 is formed by the heterodimerization of IL-23p19 and IL-12p40, while IL-12 is formed by the heterodimerization of IL-12p35 and IL-12p40. In a mouse model of colitis, neutralization of IL-23 using an IL-23-specific anti-p19 antibody significantly alleviated both emerging and established colitis, through the downstream inhibition of IL-17 expression, leading to diminished neutrophil infiltration (37). One case of severe CGD colitis treated with ustekinumab (anti-p40, the common chain of IL-12 and IL-23) showed clinical improvement but developed a severe infection (38).

Leukocyte adhesion defect (LAD) 1 is characterized by a severe periodontal disease and premature loss of teeth. Moutsopoulos et al. showed that excessive IL-17 expressing T cells in periodontal tissue is responsible for the inflammation and bone loss in human and mouse models of LAD1. These observations support targeting IL17 production through inhibition of IL-12/23 p40 (39).

#### IL-1

IL-1 is induced in response to numerous inflammatory stimuli and is the critical mediator of fever; it was previously known as “endogenous pyrogen.” Its natural antagonist, IL1RA, inhibits IL-1’s activation of its receptor and, therefore, blocks IL1-mediated inflammation. IL-1 is generated from proIL-1 by the action of the IL-1 inflammasome, mediated by NLRP3, previously known as CIAS. Gain-of-function (GOF) mutations in NLRP3 lead to autoinflammatory diseases, so-called because they do not depend on antigen-specific triggers or T or B responses. Anakinra has been used extensively in disorders of inflammation. de Luca et al. showed that inhibition of IL-1 receptor activation using the receptor blocker anakinra resulted in inflammasome inactivation, restoration of autophagy, improvement in colitis, and protection from invasive aspergillosis in p47*phox*^−/−^ mice. Studies in human subjects are essential to derive data on safety, dosage, and duration and complete efficacy of anakinra therapy in patients with CGD.

#### IL-6

IL-6 is a cytokine that induces signal transducer and activator of transcription (STAT) 3 phosphorylation and leads to fever and the acute phase response. Interestingly, it is also a myokine that is produced by muscle during activity. IL6 is elevated in RA and has been successfully targeted in that disease by tocilizumab, which inhibits downstream STAT3-mediated effects. In the disease caused by GOF STAT3 mutations, signaling is pathologically augmented, leading to fever, arthritis, rashes and lung disease. Milner et al. showed that tocilizumab led to marked improvement of peripheral arthritis and scleroderma-like skin lesions in a patient who had failed multiple other immunosuppressive therapies (40).

### Signal Blockade

#### CTLA4

CTLA4 is expressed on Treg cells and activated T cells, providing an inhibitory signal to effector T lymphocytes through its binding to CD80/CD86. Therefore, reduced CTLA4 expression leads to reduced Treg activity, which in turn leads to autoimmunity. CTLA4 deficiency is characterized by cytopenias and the triad of granulomatous brain lesions, granulomatous lung lesions and gut involvement; these manifestations can be separate or together, and either immunodeficient or autoimmune phenomena may predominate. Abatacept is a protein formed by the fusion of the Fc domain of IgG1 to the extracellular domain of CTLA4, thereby mimicking the binding of CTLA4 to CD80/CD86 and helping to tamp down autoimmunity. Lee et al. reported the effectiveness of abatacept in an adolescent girl with a mutation in CTLA4 and severe gut and other disease (41).

### T Cell-Directed Therapies

In ALPS-associated autoimmune cytopenias steroids are the first line of treatment, followed by mycophenolate mofetil (a prodrug of mycophenolic acid that inhibits inosine monophosphate dehydrogenase and suppresses T and B cells) and sirolimus [an mechanistic target of rapamycin (mTOR) inhibitor] that more effectively targets double-negative T cells (32, 42).

In other PIDs, such as CGD, methotrexate and cyclosporine have been used to control T-cell-mediated complications. Hydroxychloroquine, a moderately effective but less immunosuppressive drug, can be used as a single drug therapy or in combination with low-dose steroids. Hydroxychloroquine may enhance CLTLA4 expression through the inhibition of lysosomal CTLA4 degradation (43).

#### Janus-Associated Kinase/STAT Inhibitors

Gain-of-function mutations in STAT1 lead to sustained levels of phosphorylation of STAT1 upon stimulation, which result in increased expression of interferon-simulated genes. Autoimmunity and infection caused by STAT1 GOF mutations are thought to be the result of dysregulated T cell responses. Janus kinase inhibitors may be effective targeted treatments for long-term disease control of severely affected patients for whom hematopoietic stem cell transplantation is not available (44–46). The experience with transplantation for STAT1 GOF has so far been disappointing, suggesting that there are complex issues that will need novel approaches in terms of preparative regimen, in particular (47).

#### mTOR Inhibitors

Mechanistic Target of Rapamycin is a serine/threonine protein kinase that regulates a dizzying array of cellular and metabolic activities, especially including T cell proliferation. Conditions that constitutively or aberrantly activate the mTOR pathway lead to excess signaling, and are associated with abnormal cell proliferation and autoimmunity. Rapamycin is a small molecule that blocks mTOR activity and has found extensive clinical application in the maintenance of transplant tolerance. It has also achieved good clinical responses in patients with immune dysregulation, polyendocrinopathy, enteropathy, X-linked (IPEX) syndrome, probably related to the decrease in proliferation of T effector cells with relative preservation of Treg cells. It leads to reduction in hepatosplenomegaly and lymphadenopathy, improvement in naive T cell counts, and restitution of lymphocyte IL-2 secretion (48, 49).

In activating mutations in PIK3CD, Lucas et al. demonstrated that rapamycin restored the abundance of naive T cells, largely “rescued” the *in vitro* T cell defects and improved the clinical course (50). In *LRBA* deficiency, which is associated with impaired CTLA4 display due to LRBA’s role in CTLA4 recycling, the CTLA4 mimetic abatacept caused dramatic and sustained improvement (43).

### Apoptosis

Programmed cell death protein 1, PD-1, binds to PD-1 ligand, PDL-1, and downregulates the activity of inflammatory T cells. This interaction forms the basis of one of the immune checkpoints, a node at which immune reactivity can either be encouraged (PD-1 inhibition) or diminished (PD-1 expression). This fundamental recognition has served as the basis for the newer immune therapies in cancer, which use PD-1 inhibition to disinhibit high PD-1 expression leading to antitumor immune response. High PD-1 expression is generally associated with exhaustion of T cells, such as during chronic viral infections, and is associated with poor responses to antigen activation. Importantly, blockade of this PD-1/PD-1-ligand interaction restores antigen-specific responses *in vitro* (51). PD-1 receptor blockade increased JC virus-specific T-cell immune responses in a subgroup of progressive multifocal leukoencephalopathy (PML) patients *in vitro*, suggesting that this pathway might be important in the control of JC virus-associated PML (52).

### Miscellaneous Agents

#### Magnesium

LOF mutations in the gene encoding the X-linked magnesium transporter 1 (*MAGT1*) result in an immunodeficiency characterized by EBV infection and lymphoma (XMEN), due to impaired magnesium-dependent intracellular signaling, which is especially important in NK cell function (53). Treatment with high-dose magnesium, supplied by the oral preparation magnesium threanate has been able to restore NK activity and reduce EBV viral loads in a small number of cases.

#### Pioglitazone

Peroxisome proliferator-activated receptor γ (PPARγ) is a central mediator of metabolic responses, which has made it a target for endocrine therapies. PPARγ is involved in numerous cellular pathways, including reactive oxygen species (ROS) generation in mitochondria, fatty acid metabolism, and gluconeogenesis. Activation of intracellular PPARγ by agonists such as pioglitazone improved neutrophil ROS production and enhanced microbicidal action against *Staphylococcus aureus* in a murine CGD model (54). Apparently, pioglitazone leads to increased mitochondrial ROS, which then supplements the intracellular killing of certain microbes. Pioglitazone may also regulate other pathways, including IL-17, which is aberrantly high in CGD.

## State-of-the-Art Treatments

Chronic and refractory viral infections remain a cause of significant mortality both before and after HSCT in patients with PID. Reconstitution of T cell immunity is critical for control of viral infections. Adoptive immunotherapy with virus-specific T lymphocytes (VSTs) is an attractive option for addressing the underlying impaired T cell immunity (55). Infusion reactions are uncommon, mild and likely related to the cryopreservation additive rather than the VST themselves. To date, with limited phase 1 and 2 studies, cytokine release syndrome has not been described, though it remains a theoretical risk, particularly in patients with disseminated viral disease. And also it is unclear if the rate of GVHD in those receiving VST therapy is different from the background rate of GVHD in patients undergoing HSCT.

## Author Contributions

BM drafted and edited the manuscript. SH conceived and edited the final version.

## Conflict of Interest Statement

The authors declare that the research was conducted in the absence of any commercial or financial relationships that could be construed as a potential conflict of interest.
